# Supplementary material for: Establishment and application of a dual real-time PCR assay for differential detection of PiAdV-1 and PiAdV-2 among pigeons in China in 2022–2023
Source: PLoS One. 2025 Sep 19;20(9):e0330417. doi: 10.1371/journal.pone.0330417 (PMC12448338; doi:10.1371/journal.pone.0330417)
Supplement: Attachment — Submitted filename: Response to Reviewers.docx [file pone.0330417.s002.docx]

**To Journal Requirements:**

**1. Please ensure that your manuscript meets PLOS ONE's style requirements, including those for file naming. The PLOS ONE style templates can be found at https://journals.plos.org/plosone/s/file?id=wjVg/PLOSOne_formatting_sample_main_body.pdf and https://journals.plos.org/plosone/s/file?id=ba62/PLOSOne_formatting_sample_title_authors_affiliations.pdf.**

**Answer:** The paper format and submission documents have been revised and submitted in accordance with the journal's requirements.

2. **Thank you for submitting your work to PLOS ONE. We note that you have not mentioned the source of tissue samples from pigeons used in this study. Before we can continue with your submission, kindly mention the details in the Method section. Thank you for your attention to this request. We look forward to hearing from you.**

**Answer:** The source information of pigeon tissue samples has been added in the manuscript

3. **We noticed you have some minor occurrence of overlapping text with the following previous publication(s), which needs to be addressed:https://www.mdpi.com/1999-4915/15/6/1238 https://onlinelibrary.wiley.com/doi/10.1111/tbed.14464.
   In your revision ensure you cite all your sources (including your own works), and quote or rephrase any duplicated text outside the methods section. Further consideration is dependent on these concerns being addressed.**

**Answer:** We cited relevant literature and rephrased duplicate texts in the manuscript

4. **We note that the grant information you provided in the ‘Funding Information’ and ‘Financial Disclosure’ sections do not match.When you resubmit, please ensure that you provide the correct grant numbers for the awards you received for your study in the ‘Funding Information’ section.**

**Answer:** The information in the funding section was confirmed.

5. **Your ethics statement should only appear in the Methods section of your manuscript. If your ethics statement is written in any section besides the Methods, please delete it from any other section.**

**Answer:** We have removed the other parts of the ethics statement.

6. **We note that Figure 4 in your submission contain [map/satellite] images which may be copyrighted. All PLOS content is published under the Creative Commons Attribution License (CC BY 4.0), which means that the manuscript, images, and Supporting Information files will be freely available online, and any third party is permitted to access, download, copy, distribute, and use these materials in any way, even commercially, with proper attribution. For these reasons, we cannot publish previously copyrighted maps or satellite images created using proprietary data, such as Google software (Google Maps, Street View, and Earth).**

**Answer:** Figure 4 has been removed and the relevant content was presented as text in the manuscript.

**To **Reviewer #1:****

1. **The ICTV information provided for Adenovirus in the introduction is not up to date. It would be appropriate to change this information to its current state.**

**Answer:** Thanks for your comment. The relevant content has been modified according to the latest ICTV adenovirus information in the manuscript.
**2. The distinction between RNA and DNA viruses presented under the heading "2.5 Specificity and Sensitivity" was made incorrectly. In addition, the same abbreviation was used for both coronavirus and circovirus. It would be appropriate to correct these errors.**

**Answer:** Thanks for your advice. The distinction between RNA and DNA viruses presented have been modified. The abbreviations for coronaviruses and circoviruses have been amended in the manuscript

**3. The data presented in Table 3 and Table 5 are a bit confusing. (Example: Do PiAdV-A and PiAdV-B have the same virus dilution copies? It would be more appropriate to write the positive rate separately in Table 5.)**

**The author^,^s answer:** Thanks for your suggestion, and the contents of Tables 3 and 5 have been modified as suggested.

**To **Reviewer #2:****

1. **The exact usefulness of the differentiation of PiAdV-A and B is not clear in the introduction and the discussion. Aside from the epidemiological surveillance, how useful is the dual detection in routine farm diagnosis? Line 64-65, the authors mentioned that distinction between PiAdV-A and B is mandatory for “rapid clinical diagnosis”. Why the detection of total PiAdV is not sufficient to contain epidemics in breeding farms and what will the dual detection change in the management of epidemics in a breeding farm? The PiAdV-A prevalence appears to be very low compare to type B. Why is it relevant to differentiate between the two types in routine diagnosis?
   The authors should develop these points in the introduction and discussion.**

**The author^,^s answer:** Thanks for your comment. The explanation of the need to distinguish between PIADVA and B has been further elaborated in the introduction and discussion.

2. **Most papers about PiAdV talk about PiAdV type 1 or 2 and not variant A and B (Teske et al. 2017; Chen et al. 2024).
   Line 36. Ref 2 about “Falcon adenovirus B” refer to a paper that identified PiAdV type 2, with variant A and B that belong to the PiAdV-2. This is very confusing. The authors speak about PiAdV-1 and 2 or A and B belonging to PiAdV-2? In the phylogenetic tree of the present study (figure 5), types identified are 1 and 2 and not A and B. This is very confusing. Please revised the nomenclature.
   The author^,^s answer:** We have changed the nomenclature of PiAdV-A and PiAdV-B as suggested.
3. **Lines 52-54. Confusing. How it can be clear that the disease has been prevalent in China?**

**Line 63, the authors mentioned that the prevalence is not clear in China. If the prevalence is not known, so are the economic losses, and studies are needed to assess prevalence in China and the associated economic losses. Please revise this point.**

**The author^,^s answer:** Thanks for your suggestion. The confusing description has been revised

4. **Lines 55-59. The authors mentioned that the virus isolation is the more reliable detection method. Is it a general comment or specific to PiAdV detection? Has virus isolation been used for PiAdV diagnosis? Is there some studies showing better PiAdV detection with virus isolation than PCR? Real-time PCR is usually highly sensitive.**

**The author^,^s answer:** Thanks for your comment. This description that the virus isolation is the more reliable detection method refers to a generic description of all viruses. At present, the isolation of pigeon adenovirus in our laboratory is not go well. For this puzzling, the relevant content has been redescribed in the manuscript.

5. **Lines 57-60. Serum neutralizing antibody technology is not a common method for viral detection in laboratory, but useful for specific studies about protection of an individual or a population against a virus (seroprevalence). Seroprevalence of PiAdV neutralizing antibodies could be an interesting tool to determine the circulation of PiAdV in pigeons and for epidemiological surveillance. The added-value of seroprevalence and serum neutralizing antibody technology may be broach in the discussion and not the introduction.**

**The author^,^s answer:**Thanks for your advice. Changes have been made as your suggestion.

6. **Line 121. Please specify if testing for a single dilution was performed from one tube or from three different samples (for example three different samples of 9.48x10^9 copies of PiAdV-A). The guidelines from World Organisation for Animal Health (WOAH - “PRINCIPLES AND METHODS OF VALIDATION OF DIAGNOSTIC ASSAYS FOR INFECTIOUS DISEASES” p11) specify that “It is not acceptable to prepare a final working dilution of a sample in a single tube from which diluted aliquots are pipetted into reaction vessels, or to create replicates from one extraction of nucleic acid rather than to extract each replicate before dilution into the reaction vessels. Such ‘samples’ do not constitute valid replicates for repeatability studies”.**

**The author^,^s answer:** Due to a mistake in my language description, which led to the reviewer's doubt, some of the language in Line 126-130 (formerly Line 121) has now been revised.

7. **Line 131. About the conventional PCR. Reference 12 refer to a panadenovirus nested-PCR, and reference 13 to a PCR detecting specifically total PiAdV. Have these two methods been performed and shown the same results? Please clarify which conventional PCR has been performed in the present study. Specify the LOD of the conventional PCR assay if known.
   Table 4. Indicate more clearly in the table which one is the conventional PCR and which one is the dual.**

**The author^,^s answer:** Primers used for conventional PCR have been shown in Table 2 and more clearer description is added to Table 4.

8. **Line 147. How many negative sample (ddH_2_O) were tested?**

**The author^,^s answer:** The negative samples included 3 AIV, 1 RVA, 1 NDV, 1 PiCV, 1PiHV and 1 negative control ddH_2_O. The relevant content is redescribed in the manuscript.

9. **Lines 153-156. How many times each point of the 10-fold serially diluted standard plasmids have been tested to determine the LOD for PiAdV-A and B genome? It is frequent to see 3 to 5 testing for each dilution of the standard, to ensure the 100% positive detection for the last point. A single test for each dilution is not robust enough. Did the authors test additional dilution beyond 94.8 or 88.4 copies/µL and 0 copy /µL?
   “With a 10-fold serially diluted standard plasmids, the last dilution showing 100% response may be accepted as a conservative estimate of the lower limit of detection. A more accurate estimate may be obtained by a second stage experiment using narrower intervals in the dilution scheme focusing on the region between 100% and 0%” (i.e. in this study, between 94.8 or 88.4 and 0 copy/µL in this study) (ref WOAH- “PRINCIPLES AND METHODS OF VALIDATION OF DIAGNOSTIC ASSAYS FOR INFECTIOUS DISEASES”)**

**The author^,^s answer:** Thanks for your comment. In performing the sensitivity test, three assays were performed for each dilution of the 10-fold diluted standard, and the the relevant content has been added to the manuscript. According to instructions of WOAH-“PRINCIPLES AND METHODS OF VALIDATION OF DIAGNOSTIC ASSAYS FOR INFECTIOUS DISEASES”, a 2-fold dilution experiment below the minimum detection limit was carried out, the results showed that Piadv-1 was detected 6 times and Piadv-2 4 times out of 10 replicates performed, so the detection limits for this test remains 94.8 and 88.4 copies/µL.

**10. The authors claim that the method developed for dual detection is satisfactory, but the limit of detection is higher than the LOD of 34.6 copies /µL for PiAdV-2 PCR developed by Chen et al. 2024. This point should be discussed, in addition to the added value of the dual real-time PCR developed by the authors in comparison with the results of Chen et al. 2024.**

**The author^,^s answer:** The LOD of Chen’s article and the LOD of our experiment both belong to the 10^1^ copies/µL level, and there is no obvious difference. Chen's article focuses on the issue of YPDS and PiAdV-2, which is discussed in the discussion section of the manuscript.

**11. Line 194-196. The authors mentioned that the co-infection between PiAdV and other pathogens increase the mortality rate of pigeons. Information about co-infection with PTTV appear later, line 231. This is confusing. Is there a point to discuss about co-infection with other pathogens and PiADV-A and–B infection?
Could the type of PiAdV affect the mortality rate during such co-infection? Is it important to develop a multiplex PCR detecting those pathogens and PiAdV rather than differentiating PiAdV-A and B?**
**The authors should clarify their point about co-infection with other pathogens and their results.**

**The author^,^s answer:** Thanks for your comment. The Information about co-infection between PiAdV and other pathogens and co-infection with PTTV has been consolidated in the manuscript. In clinical tests, PiAdV-2 was detected more frequently in dead pigeons than PiAdV-1, but we did not isolate the virus successfully, so we did not conduct animal regression tests to determine their pathogenicity. It is very important to develop a multiplex PCR detecting those pathogens and PiAdV, and we will carry out this work in the next experiment.

12. **The authors mentioned a limit of detection of 10^1^ copies/µL for PiAdV-A and B in the abstract, but 94.8 copies for PiAdV-A and 88.4 for PiAdV-B are mentioned in the results and discussion. Indicate the exact LOD for each PiAdV type in the abstract and homogenise in the results. No need for 10^1^, the entire number is more comprehensible.**

**The author^,^s answer:** Thanks for your advice. The data in the summary and results have been harmonized.

13. **Line 55. Can the authors specify if the diagnosis of total PiAdV by PCR is routinely used in breeding farms, in case of pigeon diarrhoea or death?**

**The author^,^s answer:** In China, pigeon farms usually do not have their own laboratories, and when pigeons suffer from diarrhea or die, the disease material is usually sent to a laboratory that conducts pegion disease research for testing. As for PiAdV-1 or 2 or total PiAdV, it is generally carried out according to the existing detection methods in the laboratory.

14. **Line 92. Please specify what type of tissue samples (n=20) from pigeons were tested.**

**The author^,^s answer:** Thanks for your comment.The type of tissue samples (n=20) from pigeons has been supplemented in the manuscript.

15. **Lines 220-221. Is it known if a high morbidity or mortality rate of pigeons in the breeding farm of the regions with the higher PiAdV prevalence was observed?**

**The author^,^s answer:** The detection rate of PiAdV was high in the dead pigeons from the breeding farm tested by our laboratory. It can be seen that the high mortality or mortality rate of pigeons is correlated with the high prevalence of PiAdV. The relevant description has been added to the manuscript.

16. **Line 23. The dual fluorescence PCR method established in this study was used to test 500 pigeon swab samples.
    Line 37. The virion has icosahedral symmetry […] the capsid proteins mainly consist of […].
    Line 56. “has long detection cycle”, did the authors mean “is time-consuming” ?
    Lines 58-59. but these need to the more higher viral load of the sample. This need to be reworded.
    Line 138. Delete parenthesis
    Line 195. Correct pegions.**

**The author^,^s answer:** Thanks for your suggestions. The above sentences has been revised in the manuscript
