# Supplementary material for: Establishment and application of a dual real-time PCR assay for differential detection of PiAdV-1 and PiAdV-2 among pigeons in China in 2022–2023
Source: PLoS One. 2025 Sep 19;20(9):e0330417. doi: 10.1371/journal.pone.0330417 (PMC12448338; doi:10.1371/journal.pone.0330417)
Supplement: Attachment — Submitted filename: Response_to_Reviewers_auresp_2.docx [file pone.0330417.s003.docx]

**Reviewer #1: The authors have adequately addressed my comments raised in a previous round of review and I feel that this manuscript is now acceptable for publication.**

**Answer:** OK, thanks.

**Reviewer #2: The manuscript is much clearer now, and the corrections are really helpful. I spotted just two tiny little things that could be improved:
Line 238 : Correct pegions for pigeons
Figure 4 : Correct varient for variant.**

**Answer**: All corrections have been made

**Reviewer #3:
Line 17. Significance of adenoviruses in case of diarrheic diseases of pigeons. Read the latest paper from this topic, provide corrections according to it in the further parts of MS and cite it properly in the manuascript, please: doi.org/10.1016/j.virol.2025.110400.
Introduction section, and pigeon adenoviruses description: This work bases on molecular methods, so provide a brief description of PiAdV genome, please.**

**Answer**: Thanks for your comment. Related contents have been added in the manuscript.

**Line 53. Spriding of PiAdV in the loft - provide details and reference, please or delete this information.**

**Answer**: Thanks for your suggestion, and this information has been deleted in the manuscript.
**Line 64. Not true. Virus isolation is not the most common method, but it is the best method in case of proofing the pathogenicity of the virus. Rewrite this section, please.**

**Answer**: Thanks for your comment. This section has been rewrited in the manuscript.

**Line 67. "high viral loads" - What it means "high" - it is too general. Provide some examples, please.**

**Answer:** Thanks for your advice. This section has been redescribed in the manuscript. **Lines 71-74 - Does it have any practical aspect? Practical means prevention or prophylaxis programms and it's impemmentation in the pigeon flocks?**

**Answer:** Thanks for your comment. Through rapid clinical diagnosis, early detection, early isolation and early treatment can be achieved, reducing the loss of pigeon flocks. The relevant content has been added in the manuscript.

**Line 100. "Pigeon Rotavirus A" (I suppose), because other rotaviruses are also prevalent in pigeons: doi.org/10.1155/tbed/4684235. Provide the strain names used in this study, please.**

**Answer:** Thanks for your comment. I am sorry for the mistake, and the RVA strain was used in this study. Our laboratory only has RVA and no other RVs. We have made revisions in the manuscript.

**Line 102. What was the base of this suspicion? Describe it a little bit.**

**Answer:** Thanks for your comment. Liver tissue treatment was performed on 20 pigeons with clinical signs such as vomiting and diarrhea.

**Line 107. How many of each types of the swabs were investigated?**

**Answer:** Oropharyngeal swab and cloacal swab of one pigeon mixed in one tube
**Line 117. cDNA or DNA depending on the virus?**

**Answer**: The mistake has been corrected in the manuscript.
**Line 127. Missing space.**

**Answer:** All corrections have been made.

**Lines 154-158. The section "Sequencing and phylogenetic analysis" .
This section is written very scantly. Provide all sequencing details, please. Provide all details concerning the assembling the sequencing reads into contigs, sequencing errors removing and phylogenetic analysis including method for seletion nucleotide substitution algorithm. Add all information concerning software used for bioinformatic analyzes. Provide the list of accession numbers of generated under this study nucleotide sequences.**

**Answer:** Thanks for your comment. This content was supplemented with details in the manuscript.

**Line 195. The description of results of molecular screening for adenoviruses is too general. The statistical analysis of the prevalence of both pigeon aviadenoviruses could improve the results section. I reccomend to add statistical analylis to the methodology.**

**Answer:** Thanks for your comment. Appropriate additions has been added n the manuscript.

**Lines 208-209. "infected samples" - The samples were positive, not infected.**

**Answer:** Thanks for your comment. The mistake has been corrected in the manuscript.
**Lines 2010-2011 - Those results concerns PiAdV-1, PiAdV-2, or both of them?**

**Answer:** Thanks for your comment. The content in doubt has been revised in the manuscript.
**Table 5. Show results in the table separately for PiAdV-1 and PiAdV-2, please icluding statistical analysis, please.**

**Answer:** Thanks for your advice. The content has been revised in the manuscript. **Line 220. "of was" ??**

**Answer:** The mistake has been revised in the manuscript. **Fig. 4 and phylogenetic analysis: Add PP999622 and PP999623 sequences to the analysis, please.**

**Answer:** The content has been added in the manuscript.
**Line 243. This is an old data. Currently the RVA is suspected to be a causative agent of YPDS: DOI: 10.1111/tbed.13485.**

**Answer:** The content has been revised in the manuscript. **Lines 250-253. There is one more method previopusly published:
doi.org/10.1016/j.virol.2025.110400 - cite those refereences correctly, please.**

**Answer:** Thanks for your comment. The content has been added in the manuscript.
**The discussion section: Very poor discussion. Too many repetitions of the results, too less real discussion. The results of phylogenetic analysis were not discussed at all.**

**Answer:** Thanks for your comment. We have made modifications to the discussion section , and added the phylogenetic analysis in the manuscript
